# Low-dose tamoxifen treatment reduces collagen organisation indicative of tissue stiffness in the normal breast: results from the KARISMA randomised controlled trial

**DOI:** 10.1186/s13058-024-01919-1

**Published:** 2024-11-26

**Authors:** Sara Göransson, Pablo Hernández-Varas, Mattias Hammarström, Roxanna Hellgren, Magnus Bäcklund, Kristina Lång, Ann H. Rosendahl, Mikael Eriksson, Signe Borgquist, Staffan Strömblad, Kamila Czene, Per Hall, Marike Gabrielson

**Affiliations:** 1 Department of Biosciences and Nutrition, Karolinska Institutet, Huddinge, Sweden; 2 Core Facility for Integrated Microscopy, Department of Biomedical Sciences, Faculty of Health Sciences, University of Copenhagen, Copenhagen, Denmark; 3 Department of Medical Epidemiology and Biostatistics, Karolinska Institutet, Nobels väg 12A, 171 77 Solna, Sweden; 4 Department of Breast Imaging, Södersjukhuset, Stockholm, Sweden; 5 Department of Translational Medicine, Diagnostic Radiology, Lund University, Lund, Sweden; 6 Department of Clinical Sciences Lund, Oncology, Lund University and Skåne University Hospital, Lund, Sweden; 7 Department of Oncology, Aarhus University Hospital, Aarhus University, Aarhus, Denmark

## Abstract

**Background:**

Tissue stiffness, dictated by organisation of interstitial fibrillar collagens, increases breast cancer risk and contributes to cancer progression. Tamoxifen is a standard treatment for receptor-positive breast cancer and is also aproved for primary prevention. We investigated the effect of tamoxifen and its main metabolites on the breast tissue collagen organisation as a proxy for stiffness and explored the relationship between mammographic density (MD) and collagen organisation.

**Material and methods:**

This sub-study of the double-blinded dose-determination trial, KARISMA, included 83 healthy women randomised to 6 months of 20, 10, 5, 2.5, and 1 mg of tamoxifen or placebo. Ultrasound-guided core-needle breast biopsies collected before and after treatment were evaluated for collagen organisation by polarised light microscopy.

**Results:**

Tamoxifen reduced the amount of organised collagen and overall organisation, reflected by a shift from heavily crosslinked thick fibres to thinner, less crosslinked fibres. Collagen remodelling correlated with plasma concentrations of tamoxifen metabolites. MD change was not associated with changes in amount of organised collagen but was correlated with less crosslinking in premenopausal women.

**Conclusions:**

In this study of healthy women, tamoxifen decreased the overall organisation of fibrillar collagens, and consequently, the breast tissue stiffness. These stromal alterations may play a role in the well-established preventive and therapeutic effects of tamoxifen.

*Trial registration* ClinicalTrials.gov ID: NCT03346200. Registered November 1st, 2017. Retrospectively registered.

**Supplementary Information:**

The online version contains supplementary material available at 10.1186/s13058-024-01919-1.

## Introduction

Increased breast tissue stiffness leading to malfunctioning stromal-epithelial interactions is associated with an elevated risk of breast cancer [1], and both preclinical and clinical data suggest that breast architecture contributes to breast cancer initiation and progression [2–7]. The breast tissue interstitial stroma consists mainly of the extracellular matrix (ECM) component, collagen type I [8, 9]. The degree of cross-linking and formation of three-dimensional structures of type I collagen and other fibrillar collagens, a subgroup within the collagen family capable of forming fibrils, varies between individuals and dictates the mechanical properties (e.g., stiffness) of the tissue [10–12]. The organisation and stiffness of the ECM are, in turn, important for breast epithelial cell behavior, regulation of development, and normal tissue homeostasis [8, 13], and are potential contributors to elevated breast cancer risk. Breast tissue composition is reflected by the mammographic density (MD). A high MD correlates with an increase in stromal and epithelial content [14, 15] and is associated with an increased lifetime risk of breast cancer [16, 17]. The molecular mechanisms responsible for this elevated risk are not clear but may partly be linked to increased mechanical signalling from a stiffer interstitial and peri-ductal stroma, an inherent feature of high MD tissue [1, 3, 10].

The organisation of fibrillar collagens can be studied in paraffin sections using picrosirius red staining followed by circularly polarised light microscopy (PS-cPOL) [18]. The picrosirius red dye binds to collagen and enhances the natural birefringence of fibrils when viewed under circularly polarised light. The polarisation colour is dictated by the thickness and degree of crosslinking of the fibres, ranging from green for thinner and less packed immature fibres to red for thick and densely packed mature fibres [19]. Tissue birefringence, as visualised by PS-cPOL, showed a good correlation with atomic force microscopy (AFM) measurements of tissue stiffness [10, 20]. Hence, PS-cPOL can be used as a proxy for tissue stiffness, which allows relative rigidity measurements to be made in paraffin sections.

Tamoxifen, pharmacologically classified as a selective oestrogen receptor modulator (SERM), has been the standard treatment for hormone receptor-positive breast cancer for decades [21] and has been approved for primary prevention of breast cancer in several countries [22–25]. A meta-analysis of randomised tamoxifen primary prevention trials showed a 38% reduction in breast cancer incidence [26]. The IBIS-I trial revealed that 5 years of tamoxifen treatment lowered the incidence of breast cancer by at least 20 years [22]. The clinical response to tamoxifen may be connected to its conversion into the active metabolites, Z-4-hydroxy-N-desmethyl-tamoxifen (endoxifen) and Z-4-hydroxy-tamoxifen (4-OH-tamoxifen) by cytochrome P450 2D6 (CYP2D6), as they are 30–100 fold more potent than tamoxifen itself in inhibiting the oestrogen receptor (ER), and the serum concentrations of these metabolites predict long-term survival in breast cancer patients [27]. The molecular mechanisms of tamoxifen action in breast cancer prevention are not fully understood, but previous studies have shown alterations in breast tissue composition with reduced epithelial area in premenopausal women following 6 months of treatment [28], and a decrease in mammographic density (MD) has been suggested as an early marker of the therapeutic effect [29, 30]. Increased knowledge of how tamoxifen affects different aspects of breast tissue biology in healthy women is desirable, as it could help us understand which high-risk women would benefit the most from this preventive treatment.

We have previously conducted a clinical trial showing that 6 months’ exposure of tamoxifen to healthy women is sufficient to observe a significant MD decrease [31]. In the following 6-armed KARISMA tamoxifen dose-determination trial for the primary prevention of breast cancer, we found that tamoxifen in doses as low as 2.5 mg effectively decreased the MD in premenopausal women, but not in postmenopausal women [32]. Using biopsies collected within the KARISMA trial, we showed that tamoxifen at various doses changes the tissue composition and expression of proliferation markers and hormone receptors in normal breast tissue [28]. In premenopausal women, tamoxifen decreases density by altering the proportions of dense epithelial-stromal tissues and non-dense adipose tissue.

In the present study, based on the KARISMA biopsies of healthy women, we investigated how tamoxifen treatment at different doses and circulating plasma levels of tamoxifen metabolites affect the overall organisation of fibrillar collagen, and consequently the stiffness of the breast tissue. Furthermore, we explored a possible link between collagen organisation and MD.

## Materials and methods

### Trial design and participants

**Fig. 1:**
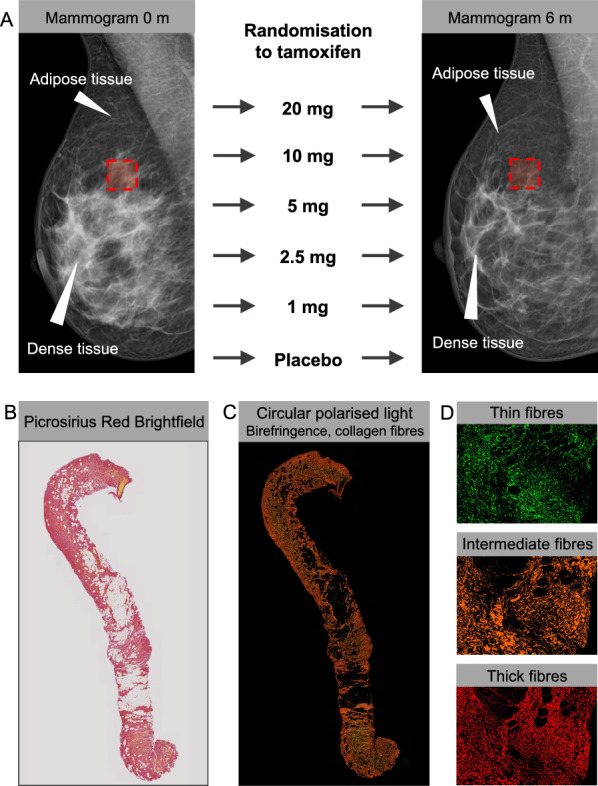
Study design. **A** Mammograms taken before and after randomisation and treatment with tamoxifen at five doses or placebo for 6 months and illustrating the site of biopsy (red square), dense glandular and stromal tissue (white), and non-dense fatty tissue (dark). **B**, **C** A biopsy stained with picrosirius red and imaged with **B** bright-field light microscopy or **C** circularly polarized light (cPOL) microscopy. **D** Representative inset images of machine-learning-based segmentation of fibre classes from cPOL images (green = thin fibres, yellow/orange = intermediate fibres, red = thick fibres)

Participants were part of the KARISMA trial for tamoxifen dose determination [32]. KARISMA is an investigator-initiated, double-blind, randomised, placebo-controlled, six-armed primary prevention trial conducted in Sweden between 2016 and 2019 (ClinicalTrials.gov ID: NCT03346200). The KARISMA trial included 1,440 healthy Swedish women without a prior history of cancer or benign breast disease, aged 40–74 years, who attended the population-based national mammography screening program. Participants were block randomised to 6 months of daily treatment, in a 1:1 ratio into six treatment arms: placebo, 1, 2.5, 5, 10, or 20 mg of tamoxifen. The KARISMA nested biopsy cohort study was conducted during the final stages of the KARISMA trial inclusion period and has been extensively described elsewhere [28, 33]. Briefly, women who were screened and invited to participate in KARISMA were invited to donate additional biopsies. The final biopsy cohort included 83 women with biopsies collected before and after treatment exposure (end-of-trial or early exit) (Fig. 1). An overall MD of ≥ 10% was required for participation in the nested biopsy cohort. MD for all participants was measured as the percent dense area using the fully automated STRATUS method [34] at baseline and end-of-study participation or exit, as previously described [32]. The average percent density of the left and right breasts at baseline was calculated and compared to the average percent density at the end of the trial period, and the density change was defined as the relative difference between these two measures. Before MD measurements and comparisons were made, images of the same breast were aligned to reduce technical differences [34]. Participants answered a web-based baseline questionnaire, and biometrics (weight, length, and body mass index (BMI)) were measured by study nurses at a clinical site. The full study protocol for the KARISMA trial can be found elsewhere [32].

### Collection of biopsies and immunohistochemistry staining

Core needle biopsies (14G) from a dense area of the breast in the upper outer quadrant were collected by a breast radiologist under ultrasound guidance. The end-of-trial or exit biopsy was collected in the same area of the breast as the first biopsy, by annotations in the baseline mammogram, and by skin inspection of the residual scar after the initial biopsy. The complete protocol for the immunohistochemistry (IHC) preparation of tissues has been reported elsewhere [28, 33]. Briefly, formalin-fixed paraffin-embedded tissues were stained and analysed at the same time point after the final participant had donated her end-of-study or exit biopsy. All retracted biopsies were assessed by a breast pathologist to confirm normal histology by hematoxylin and eosin staining (H&E) on whole biopsy-core 4-μm thick sections. IHC staining of picrosirius red was performed at HistoCenter AB Laboratories (Mölndal, Sweden), according to a previously published protocol [35]. All tissues were blinded during sample preparation and histological analyses.

### Imaging of histological slides

Sections stained with H&E were scanned using a NanoZoomer Scanner (Hamamatsu Photonics Norden, Stockholm, Sweden) generating ndpi-files. Digital whole-slide images of each section were analysed using the NDP.view 2 viewing software (Hamamatsu Photonics Norden, Stockholm, Sweden).

For sequential brightfield and circularly polarised light (cPOL) imaging of picrosirius red staining, an Axioscan 7 slide scanning microscope (Zeiss, Oberkochen, Germany) fitted with an N-Achroplan 20 × 0.45 Pol objective and an AxioCam 705c color camera was used. The effective pixel size was 0.172 µm/px and flash illumination was applied for 33 µs. After setting the camera white balance for the experimental set, a brightfield image was taken for every tile, followed by cPol acquisition by sliding it in a cube for cPol illumination, resulting in two acquisition channels. All slides were imaged systematically using the same acquisition parameters and 10% overlap between tiles. An algorithm integrated into the Zeiss Zen Blue 3.5 software enabled automatic tissue detection and hardware focus determination.

### Histological image analysis

The total biopsy tissue area (including all histological compositions) was manually selected and calculated from the H&E images using the built-in software annotation tool (Hamamatsu Photonics Norden, Stockholm, Sweden) at 5 × magnification.

To measure the total birefringence and segment fibres of different thickness classes from the acquired cPol images, we used a random-forest-based machine learning tool for pixel classification, namely the “Intellesis” module built into Zen Blue 3.5 analysis software (Zeiss, Oberkochen, Germany). The tellesis successfully segmented features in different image types [36–38]. This tool enables the classification of each pixel into human-defined categories by considering the information from both the bright-field and cPol image channels. We defined the following categories: Thick Collagen, Intermediate Collagen, and Thin Collagen, based on the red, yellow/orange, and green colours, respectively. After randomly selecting a subset of 10 images as a training subset, pixels belonging to each category were manually annotated to establish the ground truth for training the classifier. The machine learning-based model was trained iteratively, correcting deviations from the ground truth until the visual inspection of the resulting pixel classification and segmentation was satisfactory. The model was subsequently used to segment the defined categories systematically and automatically in all images in the dataset. Visual inspection of each segmented image was performed as quality control. Example images of the three classes of segmented fibres, as well as a colour palette for the segmented layers, are shown in Supplementary Fig. 1. After segmentation was performed, the area (µm^2^) of the different channels was extracted and handled as csv files. Segmented patches with areas smaller than five pixels were excluded from the downstream analysis. The model is available upon request.

### Tamoxifen metabolite measurements in plasma

Blood sampling was performed at the end of the trial or early exit and analysed within 3 months of blood collection, as previously described [39]. In brief, plasma concentrations of tamoxifen, endoxifen, 4-hydroxy-tamoxifen, and N-desmethyl-tamoxifen were measured using protein precipitation and liquid chromatography, followed by ultra-performance liquid chromatography-tandem mass spectrometry (TSQ Quantiva with Dionex Ultimate 3000 system, Thermo Scientific, Waltham, MA) using a method developed in the Karolinska University Hospital Clinical Pharmacology laboratory [40].

### Statistical analysis

Collagen or histological analyses were missing for three participants and the final cohort included 80 women with biopsies from both time points (Table 1). Differences in baseline characteristics across the randomisation groups were tested using univariate ANOVA and F-test. The a priori defined primary outcome of this study was to test the effects of tamoxifen on organised collagen compositions. The difference in percentage expression describes the absolute difference in percentage unit change. Due to low numbers, the randomisation groups were merged into 0 to 1 mg/day (“null-dose,” n = 23), 2.5 to 5 mg/day (“low-dose,” n = 27 and 10 to 20 mg (“high-dose”, n = 30) of tamoxifen, as previously done [28, 41]. Univariate ANOVA and F-test were used to compare percent unit change between null-dose and low- or high-dose tamoxifen. Trends across groups were tested with linear regression, and distributions between fibre types within randomisation groups by multivariate general linear models comparing differences between factors and F test for variance across factors. Associations between circulating metabolite levels and changes in collagen organisation were tested using linear regression and unstandardised beta coefficients. To test for associations between tamoxifen-induced MD change and collagen composition, we included all women treated with tamoxifen (1–20 mg), by linear regression and unstandardised beta coefficients, and distributions between fibre types by quartiles of MD change by multivariate general linear models comparing differences between factors, and F test for variance across factors.

**Table 1:** Baseline characteristics of the study population (n = 83) within the KARISMA clinical trial of low-dose tamoxifen

Characteristic	Overall
Age, mean years (SD)	51.9 (10.0)
BMI, mean kg/m^2^ (SD)	25.4 (4.1)
Menopausal status, n (%)	
Premenopausal, n (%)	48 (57.8)
Postmenopausal, n (%)	35 (42.2)
Mammographic density at baseline, mean cm^2^ (SD)	42.0 (24.1)
Stromal area at baseline, mean % (SD)	58.6 (20.0)
Epithelial area at baseline, mean % (SD)	6.7 (6.6)
Adipose area at baseline, mean % (SD)	34.6 (21.0)
Organised collagen composition, total area of biopsy	
Birefringence total area, mean % (SD)	46.6 (20.4)
Thin fibre total area, mean % (SD)	10.3 (5.6)
Intermediate fibre area, mean % (SD)	16.1 (9.7)
Thick fibre area, mean % (SD)	20.2 (10.9)
Missing, n (%)	3 (3.6%)
Organised collagen composition, proportions of birefringence	
Thin fibre area of birefringence, mean % (SD)	29.0 (22.7)
Intermediate fibre area of birefringence, mean % (SD)	30.8 (12.7)
Thick fibre area of birefringence, mean % (SD)	40.2 (12.6)
Missing, n (%)	3 (3.6%)

*BMI* Body mass index, *SD* Standard deviation

Analyses were conducted for all samples together (adjusted for menopausal status) and were stratified by menopausal status. *P* values were two-sided and considered statistically significant if < 0.05. All analyses were performed using SPSS version 28 (IBM Corporation).

## Results

### Baseline characteristics

The average age of the entire population was 51.8 years, and 57.8% of the women were premenopausal (Table 1). By measurements of the total breast tissue area from baseline biopsies, the average birefringent area was 46.7%. By proportions of birefringence, Thin and intermediate fibres constituted approximately 30% each of the total birefringence, and thick fibres constituted remaining 40%. There were no significant differences in mammographic density or tissue composition characteristics (stromal, epithelial, or adipose tissue) between the merged randomisation groups at baseline. Regarding the relative total birefringent areas and respective fibre areas at baseline, the high-dose group (10–20 mg) had a somewhat larger birefringent area than the null-dose group (0–1 mg) (mean 51% vs. 39%, *P* = 0.040) and larger thick fibre area 23% vs. 16%, *P* = 0.034). There was no significant difference between the total thin and intermediate fibre areas.

### Effects of tamoxifen on the collagen of the normal breast

**Fig. 2:**
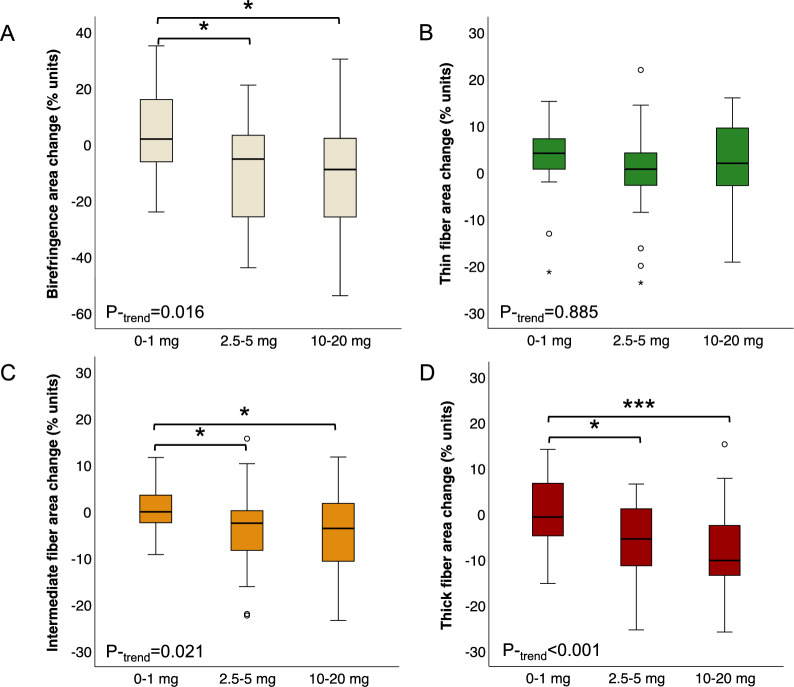
Low-dose tamoxifen treatment reduces the amount of thick and densely organised collagen in the breast. **A**–**D** Change in area of **A** total birefringence (all organised collagen), **B** thin collagen fibres, **C** intermediate collagen fibres, or **D** thick collagen fibres by merged treatment arms of tamoxifen or placebo. Percentages are relative to the total biopsy area, and all analyses were adjusted for menopausal status. *P*_-trend_ for linear trends across all groups. *P < 0.05 and ***P < 0.001 between indicated groups

When investigating the effect of tamoxifen on fibrillar collagens using PS-POL, we found that the relative amount of birefringent, organised collagen in relation to the total biopsy tissue area decreased already at 2.5 mg of tamoxifen (Fig. 2A). This was the result of a loss of thicker and more densely packed collagen fibres, as seen from the decrease in the relative amount of yellow and red signals in the polarised light images (Fig. 2B–D). In contrast, the thin fibres did not exhibit a significant change. A reduction in thicker collagen fibres after tamoxifen treatment was observed irrespective of menopausal status (Supplementary Fig. 2).

### Effects of tamoxifen and plasma-level endoxifen on collagen organisation of the normal breast

**Fig. 3:**
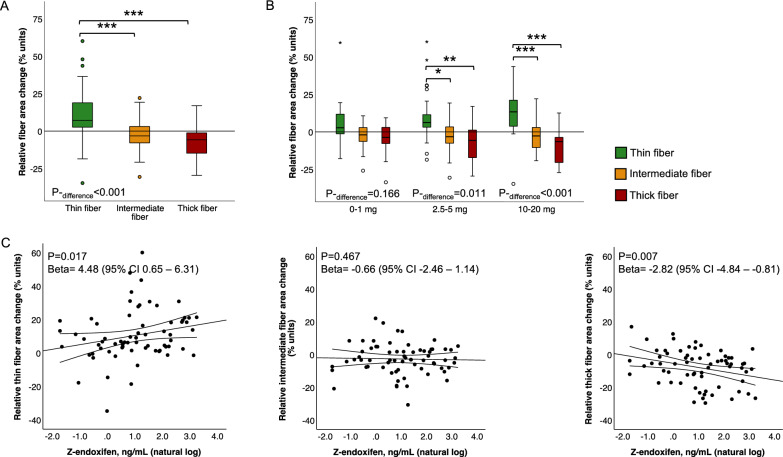
The collagen organisation in the breast is decreased by tamoxifen treatment and correlates to plasma-level endoxifen. **A**, **B** Change in area of different fibre classes within the organised collagen compartment in **A** all women treated with tamoxifen (1–20 mg) or **B** treated with tamoxifen or placebo. **C** Association between circulating endoxifen concentrations and change in area of (left) thin collagen fibres (middle) intermediate collagen fibres and (right) thick collagen fibres. Area changes were relative to the area of total birefringence, and all analyses were adjusted for menopausal status. *P < 0.05; **P < 0.01, and ***P < 0.001 between indicated groups. *P*_-difference_ for differences in distributions between all groups. Lines represent fitted regressions with mean 95% confidence interval. *P* for linear regression, beta for unstandardized beta coefficients, and 95% confidence interval on natural logarithmic transformed variable and non-transformed values

Next, we evaluated the fraction of different fibre thicknesses in relation to the total birefringent collagen to detect a possible effect of tamoxifen on collagen crosslinking. The breast tissue collagen was remodelled from thicker, more bundled fibres towards thinner, less crosslinked fibres in study participants treated with tamoxifen (1–20 mg) (Fig. 3A). A decrease in collagen organisation and crosslinking was observed at low-dose tamoxifen (2.5–5 mg) but was more pronounced at higher doses (10–20 mg) (Fig. 3B). A reduction in collagen organisation was observed, irrespective of menopausal status (Supplementary Fig. 3). Notably, the degree of collagen reorganisation correlated with circulating concentrations of endoxifen (Fig. 3C), as well as with plasma concentrations of tamoxifen itself and additional secondary metabolites 4-OH-tamoxifen and N-DM-tamoxifen (Supplementary Fig. 4). Plasma concentrations were positively correlated with thin fibres and negatively correlated with the thickest, most bundled fibres (Fig. 3C and Supplementary Fig. 4).

### Collagen reorganisation after tamoxifen treatment in relation to mammographic density change

We first investigated the association between collagen organisation and mammographic density at baseline and detected a correlation between the total percentage of MD and collagen birefringence within the total tissue area (Supplemental Fig. 5). When testing the association between percentage MD and the percentage area of different fibre thicknesses, only the intermediate and thick fibres remained positively correlated with MD (Supplemental Fig. 5). The findings were similar in both the premenopausal and postmenopausal women. This suggests that there is a link between MD and collagen organisation at baseline.

**Fig. 4:**
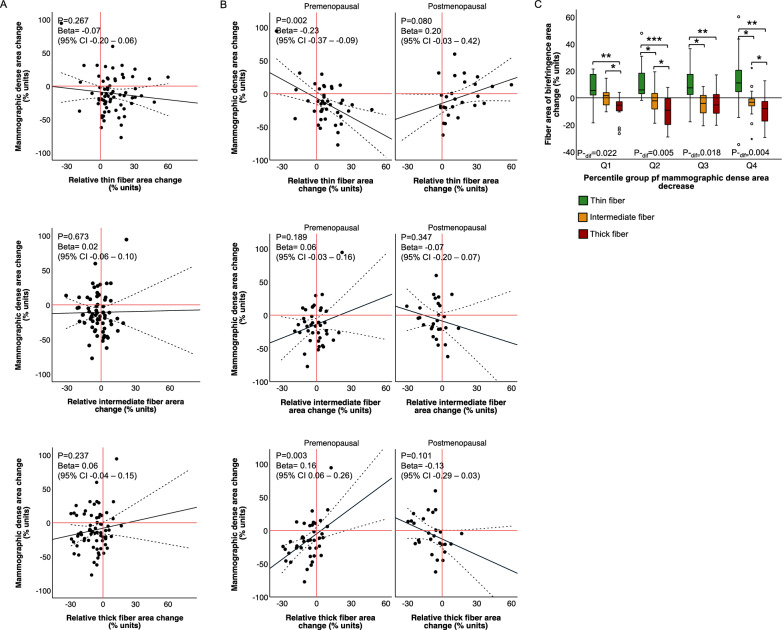
Collagen re-organisation after tamoxifen treatment is independent of mammographic density change. **A**, **B** Association between MD change and change in (upper) thin fibre area, (middle) intermediate fibre area, or (lower) thick fibre area in **A** all women treated with tamoxifen (1–20 mg) and **B** stratified by menopausal status. **C** Change in area of different fibre classes by quartiles of tamoxifen-induced (1–20 mg) MD change. Median MD change: Q1: − 44.9%, Q2: − 21.1%, Q3: − 6.6%, and Q4: + 17.5%. Changes in fibre areas were relative to the area of birefringent collagen in all comparisons. **A** and **C** are adjusted for menopausal status. Lines represent fitted regressions with mean 95% confidence interval. *P* for linear regression, beta for unstandardized beta coefficients, and 95% confidence interval on non-transformed values. *P < 0.05; **P < 0.01, and ***P < 0.001 between indicated groups. *P*_-dif_ for differences in distribution among all groups

Subsequently, we investigated the association between tamoxifen-induced changes in collagen organisation and MD. We detected no overall correlation between change in MD and change in total birefringence relative to the total biopsy area (Supplementary Fig. 6) or change in area of any of the individual fibre thickness classes within the birefringent collagen (Fig. 4A). Knowing that the decrease in density was confined to premenopausal women [32], we tested the correlation stratified by menopausal status. We found a significant correlation between density decrease and loss of the thickest collagen fibres in premenopausal women and between density decrease and an increase in the thinnest fibres (Fig. 4B). However, no correlation was found between MD changes and changes in any of the fibre types in postmenopausal women (Fig. 4B). This indicates that remodelling of collagen organisation could occur without a concomitant measurable decrease in MD. Indeed, when testing the significance of collagen reorganisation stratified by the extent of tamoxifen-induced density change, we detected significant reorganisation of collagen fibres in all quartiles of density change (Fig. 4C).

## Discussion

To our knowledge, this is the first study to show that tamoxifen reduces interstitial collagen organisation in the breast tissue of healthy women. We detected a decrease in the area covered by intermediate and thick collagen fibres in relation to the total biopsy area after 6 months of tamoxifen treatment. Furthermore, within the population of birefringent collagen, the thickest and most bundled collagen fibres were partially replaced by thinner immature collagen fibres, indicating a decrease in collagen crosslinking and a consequential reduction in tissue stiffness. Moreover, the effect was observed already at 2.5 mg of tamoxifen, a dose well below the clinical standard treatment of 20 mg for adjuvant breast cancer therapy. The effect on collagen remodelling was associated with circulating concentrations of tamoxifen and active metabolites. Plasma levels of tamoxifen and active metabolites were positively correlated with the thinnest fibres and negatively correlated with the thickest fibres, indicating a dose-dependent decrease in collagen crosslinking rather than an overall reduction in collagen synthesis and deposition.

Stromal stiffening due to remodelling of interstitial collagen is a characteristic feature of breast malignancies that likely contributes to tumour progression [13]. Increased stiffness and greater organisation of the stroma are associated with increased mammary epithelial cell proliferation [3] and stem-progenitor cell frequency [4], all of which may collectively or individually contribute to breast cancer risk. Pregnancy-induced long-term protection from breast cancer is linked to an abundant but unorganised, low-stiffness stroma [44], and the short-term postpartum increased risk coincides with a denser and more crosslinked collagen at the time of involution [9]. It is hypothesised that it is the organisation, and not the amount of collagen per se, that infers breast cancer risk. Thus, it is not surprising that targeting the stromal compartment and the stiffness-induced signalling is becoming an increasingly attractive therapeutic strategy for solid cancers [42, 43]. Hence, we speculated that the tamoxifen-induced decrease in collagen crosslinking detected in our study is part of the well-established preventive and therapeutic effects of tamoxifen.

The molecular details of how tamoxifen decreases collagen organisation in the breast have not been investigated here, but preclinical data from a mouse pancreatic cancer model suggest that tamoxifen reduces collagen content and organisation in a lysyl oxidase-like 2 (LOX-L2)-dependent manner [45]. Furthermore, via its activation of the G protein-coupled oestrogen receptor (GPER), tamoxifen hampered the activity of both pancreatic [46] and hepatic [47] stromal stellate cells via activation of the G protein-coupled oestrogen receptor, leading to a less organised extracellular matrix. Intriguingly, a pilot study using affinity proteomic profiling of plasma proteins from 88 randomly selected KARISMA clinical trial study participants treated with placebo or 20 mg tamoxifen indicated effects on collagens and matrix remodelling enzymes (unpublished data). Future studies will investigate whether the decrease in collagen organisation in breast tissue detected in this study stems from the deactivation of resident stromal cells and/or the lower activity of matrix remodelling enzymes.

High-MD tissue has more organised collagen and is stiffer than low-MD tissue; however, the nature of the correlation between collagen organisation and MD is not entirely clear. Some studies have shown an association between collagen birefringence and MD [3, 10], while others have indicated that collagen microarchitecture, that is, collagen birefringence in a biopsy, may not translate into macroscopic mammographic features [48]. In this study, we detected a correlation between the thickest and most bundled fibres and MD at baseline, irrespective of menopausal status. We also observed an association between decreased MD and reduced crosslinking, and hence tissue stiffness, in premenopausal women following tamoxifen treatment, strengthening the idea of a linkage between tissue stiffness and MD. However, we found no association between MD change and change in the birefringent area following tamoxifen treatment, independent of menopausal status. This suggests that although some women experience both reduction in MD and collagen crosslinking following tamoxifen treatment, remodelling of the interstitial collagen is not necessarily reflected at the level of MD by radiologic imaging. This is especially apparent in postmenopausal women, where no decrease in MD following tamoxifen treatment was observed; however, a decrease in collagen crosslinking was still evident and correlated with circulating concentrations of tamoxifen and active metabolites. We hypothesize that this indicates that collagen reorganisation precedes the MD decrease, that is, the collagen alterations detected in postmenopausal women will eventually lead to a MD decrease in a longer treatment period. However, it is also a possibility that tamoxifen induces changes in the stroma that are not translated to MD change in certain women. Based on prior studies linking breast stromal architecture with cancer risk and progression [1–7], a decrease in tissue stiffness could hypothetically be sufficient to induce a preventive effect of tamoxifen, regardless of the outcome on MD. Future clinical studies should further explore this notion.

Interestingly, a recent study found that tissue stiffness, measured using Magnetic Resonance elastography (MRE), is an independent predictor of risk status in women with dense breasts [49]. This suggests that different high MD tissues may have diverse mechanical properties that affect the risk of developing breast cancer and implies that MD and tissue stiffness are not strictly correlated, even though high MD tissues are stiffer than low MD tissues. Together with the data presented here, this indicates that women with both dense and stiff breasts may benefit from preventive tamoxifen treatment. Notably, textural features based on digital mammograms improve risk prediction compared to percentage density measurements alone [50]. In addition, the rationale for developing deep learning mammography-based models to refine breast cancer risk assessment assumes that there is subtle and clinically relevant information in the images that is not discernible to the human eye but can be unveiled using artificial intelligence (AI) [51, 52]. The recently presented deep learning model, Spatially Transformed Inferential Force Map (STIFMap), can predict tissue stiffness from collagen morphology in immunofluorescence images [53]. Determining tissue stiffness from mammograms using AI may improve the prediction of risk and therapeutic response.

The KARISMA trial provides unique possibilities for studying the changes in normal breast tissue in cancer-free women after tamoxifen treatment at different doses. Although the study was relatively small, it remains the largest of its kind. Nonetheless, the restricted number of participants caused statistical restraints, thus necessitating the merging of randomisation groups. This approach of merging randomization-groups is supported by previous findings of the KARISMA trial [28, 33, 41]. In addition, the high-dose group had a somewhat greater birefringent area than the null-dose group at baseline, possibly influencing the overall magnitude of the associated change after tamoxifen administration. However, this effect is likely modest, as the analyses compared the relative changes within the birefringent area. Furthermore, the intention was to study stiffness, which has not been measured directly, even though the proxy used herein is reasonable. Strengths of the study is the possibility of analysing paired samples collected before and after therapy, and that the ultrasound-guided biopsies were targeted to the same area of the dense part of the breast at baseline and at the end of the study. In addition, the density decrease observed in our samples was representative of the density decrease observed in the entire KARISMA trial population [32].

## Conclusion

We found that low- and high-dose tamoxifen inhibited collagen crosslinking, which was reflected in a decrease in tissue stiffness. Moreover, alterations in collagen organisation correlated with circulating concentrations of tamoxifen and its metabolites. These stromal alterations and the subsequent decrease in tissue stiffness could affect breast cancer risk and may play a role in the established preventive and therapeutic effects of tamoxifen.

## Supplementary Information

Additional file 1.

## Data Availability

The data that support the findings of this study are available from the corresponding author and www.karmastudy.org upon reasonable request.
