# Supplementary material for: Low-dose tamoxifen treatment reduces collagen organisation indicative of tissue stiffness in the normal breast: results from the KARISMA randomised controlled trial
Source: Breast Cancer Res. 2024 Nov 26;26:163. doi: 10.1186/s13058-024-01919-1 (PMC11590516; doi:10.1186/s13058-024-01919-1)
Supplement: Supplementary file 1 — Additional file 1. [file 13058_2024_1919_MOESM1_ESM.docx]

# Low-dose tamoxifen treatment reduces collagen organisation indicative of tissue stiffness in the normal breast: Results from the KARISMA randomised controlled trial

**Supplemental Figure S1.**

**Supplemental Figure S1.**

**A** Representative cPOL inset image from a biopsy. **B** Images showing (left) thin, (middle) intermediate, and (right) thick fibres as segmented from the image in **(A)** using a random-forest-based machine learning tool (Intellesis) for pixel classification. **C** To illustrate the efficiency of the machine learning- based segmentation, colour palettes were obtained from each of the segmented categories. The digital resolution of the image was reduced by applying 5x5 binning, and the resulting TIFF images were imported into Inkscape, an open-source vectorial graphics editor. The pixel colours from each individual segmented layer (thick, intermediate and thin collagen fibres) were extracted by using the Tracing tools in the software, and classified according to colour code (using hue, saturation and lightness). The 30 most frequent pixel colours were used to populate a colour palette for each segmented layer.

**Supplemental Figure S2.**

### Supplemental Figure S2. Low-dose tamoxifen treatment reduces the amount of thick and densely organized collagen in the breast.

### A-D Change in area of (A) total birefringence (all organized collagen), (B) thin collagen fibres, (C) intermediate collagen fibres or (D) thick collagen fibres by merged treatment arms of tamoxifen or placebo, all stratified by menopausal status. Percentages are relative to total biopsy area. *P*_-trend_ for linear trend across all groups. * for *P*<0.05 and ** for *P*<0.01 for comparisons of change between baseline and end-of-study, by general linear model and paired analyses of within-subject factors.

**Supplemental Figure S3**

**Supplemental Figure S3. The collagen organization in the breast is decreased by tamoxifen treatment in both premenopausal and postmenopausal women**

**A, B** Change in area of different fibre classes within the organized collagen compartment in (**A**) all women treated with tamoxifen (1-20 mg) stratified by menopausal status, or (**B**) by merged treatment arms of tamoxifen or placebo, stratified by menopausal status. Area changes are relative to area of total birefringence and all analysis are adjusted for menopausal status. * for *P*<0.05, ** for *P*<0.01, and *** for *P*<0.001 between indicated groups. *P*_-difference_ for difference in distributions between all groups.

**Supplemental Figure S4**

**Supplemental Figure S4: The collagen re-organization in the breast correlates to plasma-levels of tamoxifen metabolites**

Association between circulating tamoxifen **(A)** 4-OH-tamoxifen **(B)** or N-DM-tamoxifen **(C)** concentrations and change in area of (left) thin collagen fibres (middle) intermediate collagen fibres and (right) thick collagen fibres. Area changes are relative to area of total birefringence and all analysis are adjusted for menopausal status. Lines represent fitted regressions with mean 95% confidence interval. *P* for linear regression and beta for unstandardised beta coefficients and 95% confidence interval on natural logarithmic transformed variable and non-transformed values.

**Supplemental Figure S5**

**Supplemental Figure S5: Association between mammographic density and fiber composition at baseline.**

Linear association between baseline **(A)** total birefringence (all organised collagen), **(B)** thin collagen fibres, **(C)** intermediate collagen fibres or **(D)** thick collagen fibres, relative to total biopsy area for all women combined and stratified by menopausal status. Analysis with all women combined are adjusted for menopausal status. Lines represent fitted regressions with mean 95% confidence interval. *P* for linear regression and beta for unstandardised beta coefficients and 95% confidence interval on natural logarithmic transformed variable and non-transformed values.

**Supplemental Figure S6**

### Supplemental Figure S6: Birefringence area change after tamoxifen treatment is independent of mammographic density change

**A-B** Association between MD change and change in birefringence area in (**A**) all women treated with tamoxifen (1-20 mg) and (**B**) stratified by menopausal status. A is adjusted for menopausal status. Lines represent fitted regressions with mean 95% confidence interval. *P* for linear regression and beta for unstandardised beta coefficients and 95% confidence interval on non-transformed values.
